# Dr. James W. White

**Published:** 1891-06

**Authors:** 


### DR. JAMES W. WHITE.

We are only able to announce the death of Dr. White in this number. It took place at his residence in this city (Philadelphia) on the morning of the 27th, from an apoplectic attack. He was actively engaged in his office up to a late hour the evening previous. A full account of his life-work will appear in the next number.

